# Clinical and MRI Differences Between Patients With Subacute Combined Degeneration of the Spinal Cord Related vs. Unrelated to Recreational Nitrous Oxide Use: A Retrospective Study

**DOI:** 10.3389/fneur.2021.626174

**Published:** 2021-02-02

**Authors:** Han Gao, Weishuai Li, Jing Ren, Xiaoyu Dong, Ying Ma, Dongming Zheng

**Affiliations:** Department of Neurology, Shengjing Hospital of China Medical University, Shenyang, China

**Keywords:** nitrous oxide, subacute combined degeneration of the spinal cord, vitamin B12, MRI, substance abuse

## Abstract

**Objective:** To explore the clinical and imaging characteristics of subacute combined degeneration of the spinal cord (SCD) related to recreational nitrous oxide (N_2_O) use.

**Methods:** Clinical and imaging data were retrospectively collected from patients with SCD related to recreational N_2_O use who were diagnosed and treated at Shengjing Hospital of China Medical University from January 2016 to June 2020. The clinical and imaging features of patients with recreational N_2_O-related SCD were compared with those of patients with N_2_O-unrelated SCD, who were diagnosed and treated during the same period of time.

**Results:** The study enrolled 50 patients (male/female: 22/28, age: 21.4 ± 4.7 years) with N_2_O-related SCD and 48 patients (male/female: 27/21, age: 62.0 ± 11.4 years) with SCD unrelated to N_2_O use. The most common signs/symptoms of the patients in both groups were limb numbness and weakness and unsteady gait, but the incidence of limb weakness, unsteady gait, disorders of urination and defecation, anorexia, reduced deep sensation in lower limbs, ataxia, and positive Babinski sign were lower in the N_2_O-related SCD group than those in the N_2_O-unrelated SCD group (*P* < 0.05). The functional disability rating score of patients in the N_2_O-related SCD group (median: 3, IQR: 2–5) was also significantly lower than the score in the N_2_O-unrelated SCD group (median: 5, IQR: 4–7) (*P* < 0.05). The serum vitamin B12 level was significantly lower in the N_2_O-unrelated SCD group (median: 96 pg/mL, IQR: 50–170 pg/mL) than the level in the N_2_O-related SCD group (median: 218 pg/mL, IQR:121–350 pg/mL) (*P* < 0.05), while both groups had similarly increased levels of homocysteine (*P* > 0.05). Compared with the N_2_O-unrelated SCD patients, more patients with N_2_O-related SCD had abnormal spinal magnetic resonance imaging (MRI) scans (80.0 vs. 64.2%). The patients with N_2_O-related SCD also had wider spinal lesions on sagittal MRI (5.3 ± 0.8 mm vs. 4.2 ± 1.0 mm), fewer spinal segments with lesions (median: 5, IQR: 4–6 segments vs. median: 6, IQR: 5–7.5 segments), and a higher incidence of the inverted V sign on axial MRI (72.0 vs. 31.2%) (all *P* < 0.05).

**Conclusion:** The recreational use of N_2_O has become an important cause of SCD in young patients. Compared with the N_2_O-unrelated SCD patients, the N_2_O-related SCD patients had less severe clinical presentations, less obvious decrease in serum VB12 levels, and more obvious MRI changes.

## Introduction

The recreational use of nitrous oxide (N_2_O) has a long history in Western countries (1), and the popularity of N_2_O as a recreational drug has increased over the last 20 years. The 2016 Global Drug Survey found it to be the seventh most popular recreational drug worldwide (2). Although N_2_O has been used in China for 10 years or less, its use has recently became widespread throughout the country because of the absence of laws to regulate its use (3). Many studies have shown that chronic exposure to N_2_O can induce various neurological conditions, including myelopathy and peripheral neuropathy (4–6). Located at the largest medical center in northeast China, our team has treated more than 100 patients with neurological conditions caused by the recreational use of N_2_O over the past 5 years. The most common condition is subacute combined degeneration of the spinal cord (SCD). Although early diagnosis and treatment are key to good outcomes, many young patients who use N_2_O are reluctant to divulge their exposure history. Thus, it is important for clinicians to be aware of the clinical and imaging features of SCD induced by N_2_O use to avoid missing patients suspected of its use. In this retrospective study, we aimed to identify the clinical and imaging characteristics of patients with N_2_O-related SCD. We analyzed and compared the clinical and magnetic resonance imaging (MRI) data of patients with N_2_O-related SCD with the data of patients who were diagnosed and treated during the same period of time with classical SCD that was unrelated to N_2_O use at our center.

## Materials and Methods

### General Data

Patients with SCD related to the recreational use of N_2_O and patients with SCD without an exposure history of N_2_O who were diagnosed and treated in the Department of Neurology, Shengjing Hospital of China Medical University from January 2016 to June 2020 were retrospectively enrolled.

The inclusion criteria for the patients with N_2_O-related SCD were as follows: (1) history of N_2_O inhalation without the concomitant use of other addictive agents and narcotics; (2) subacute or acute onset with signs/symptoms of impaired posterior and/or lateral funiculus of the spinal cord after N_2_O use; (3) decreased serum Vitamin B12 (VB12) level or positive response to VB12 treatment; (4) no other causes of VB12 deficiency (e.g., long-term vegetarian diet, gastrointestinal surgery) or past history of macrocytic anemia or VB12 deficiency before exposure to N_2_O; (5) no other types of spinal cord conditions. The inclusion criteria for N_2_O-unrelated SCD patients were as follows: (1) relevant causes for VB12 deficiency (e.g., long-term vegetarian diet, digestive system diseases, gastrointestinal surgery); (2) subacute or acute onset with the signs/symptoms of impaired posterior and/or lateral funiculus of the spinal cord; (3) decreased serum Vitamin B12 level or positive response to VB12 treatment; (4) no exposure history to N_2_O; (5) no other spinal cord problems.

Detailed clinical data were collected, such as demographic data, clinical manifestations, functional disability rating scores (FDRS), laboratory test results, and spinal MRI findings.In the laboratory tests, serum homocysteine was detected by colorimetry and serum VB12 and folic acid were determined by chemiluminescence immunoassay. Blood routine was detected by Sysmex XN9000 automatic hematocyte analyzer. This study was approved by the Ethics Committee of Shengjing Hospital, according to the Helsinki Declaration (2013).

### Functional Disability Rating Scores for Patients With SCD

We evaluated the clinical signs/symptoms of the 2 groups of the patients based on the FDRS. The specific items that were assessed and the assessment scores are described here. Gait was rated as normal (0), unable to maintain the Romberg position (1), impaired, but able to walk unsupported (2), substantial support required for ambulation (3), or wheelchair- or bed-bound (4). Sensory disturbance (hypesthesia, dysesthesia, or position sense impairment) was rated as absent (0), impairment only in toes and fingers (1), impairment in ankles and wrists (2), or impairment in upper arms and legs (3). Mental function was rated as normal (0), intellectual or behavioral impairment requiring no social support (1), partial dependence for activities of daily living (2), or complete dependence for all activities of daily living (3). Neuropathy (assessment of reflexes) was rated as absent (0), loss or reduction of deep tendon reflexes of the ankle (1), loss or reduction of deep tendon reflexes of the patella (2), or loss or reduction of deep tendon reflexes of the arms (3). Pyramidal tract functioning was rated as normal (0), positive Babinski sign (1), spastic paraparesis (2), or spastic tetraparesis (3). The minimum overall score was 0 and the maximum was 16. Higher scores represented increased severity of neurological impairment (7).

### Spinal MRI Data

**Figure 1:**
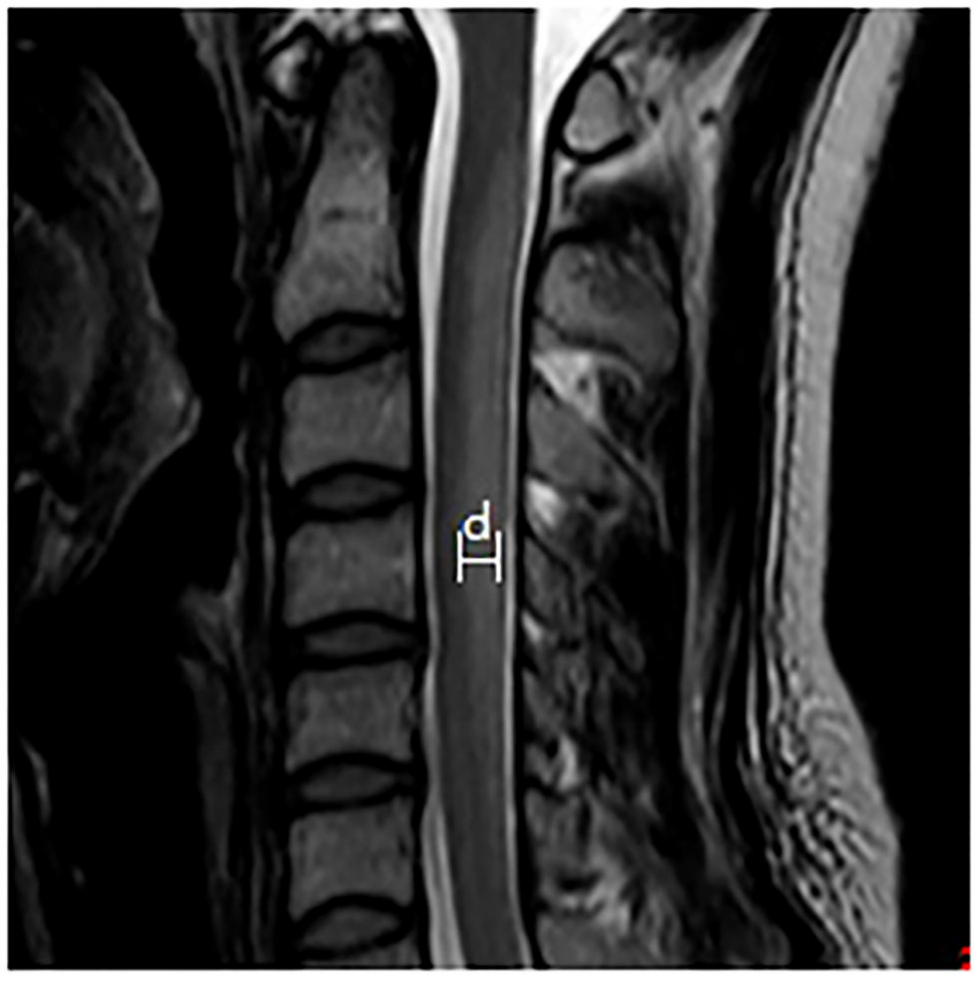
Schematic diagram of the lesion width that was measured. The maximum width between the anterior-posterior margin of the lesion in the midsagittal T2WI image is measured to provide the width of the lesion.

Spinal MRI was performed by a Trio 3.0 T MRI scanner (Siemens, Germany). The scanning sequences included sagittal and axial T1 weighted imaging (T1WI), T2WI, and fluid attenuated inversion recovery (FLAIR) sequences. The extent of spinal lesions was measured in the T2WI sagittal images (turbo spin-echo sequence, TR/TE, 2,500/90 ms; matrix, 224 × 162; and slice thickness, 3 mm), and was assessed according to the number of affected spinal segments, lesion widths (measured as shown in Figure 1), and the proportion of lesions affecting both the cervical and thoracic spinal cord. The proportion of lesions showing the inverted V sign in the T2WI axial images was also calculated.

### Statistical Analysis

Quantitative data were presented as means and standard deviation (SD) (x ± s). Intergroup comparisons were performed by the *t*-test. Qualitative data were described as counts or percentages, and intergroup comparisons were performed by the chi-squared test. Non-normally distributed data were expressed as medians and interquartile range (IQR) and analyzed by the Mann-Whitney *U*-test. *P* < 0.05 was considered statistically significant. Statistical analysis was performed using SPSS 24.0 (SPSS Inc., Chicago, IL, United States).

## Results

### Demographics and Clinical Features

The study enrolled 50 (male/female: 22/28) and 48 (male/female: 27/21) patients in the N_2_O-related SCD group and the N_2_O-unrelated SCD group, respectively (Table 1). The difference between the proportions of male and female patients in the 2 groups was not significant. The mean age (21.4 ± 4.7) years and median onset-to-admission time (median: 30, IQR: 14–60 days) of the N_2_O-related SCD group were significantly lower than those of the N_2_O-unrelated SCD group (62.0 ± 11.4 years; median: 105, IQR: 30–365 days; *P* < 0.001). At admission, the most common clinical manifestations in both groups were limb numbness and weakness, and unsteady gait. Other neurologic manifestations included urination and defecation disorders, impaired memory, abnormal limb movements, anorexia, and abnormal skin pigmentation. The incidence rates of limb weakness, unsteady gait, urination and defecation disorders and anorexia in the N_2_O-related SCD group were significantly lower than those in the N_2_O-unrelated SCD group. Patients in the N_2_O-related SCD group also showed fewer positive signs such as impaired deep sensation and a positive Babinski sign on neurological examination than patients in N_2_O-unrelated SCD group (*P* < 0.05). Accordingly, the difference between the FDRS of the 2 groups was significant (N_2_O-related SCD, median: 3, IQR: 2–5 vs. N_2_O-unrelated SCD, median: 5, IQR: 4–7), indicating that on admission, the patients with N_2_O-related SCD had milder disease than the patients with N_2_O-unrelated SCD.

**Table 1:** Demographics, clinical, and investigations data in N_2_O group and non-N_2_O group patients.

	**N_**2**_O-related SCD group (*n* = 50)**	**N_**2**_O-unrelated SCD group (*n* = 48)**	***P-*value**
Male: Female	22:28	27:21	0.225
Age (years)	21.4 ± 4.7 (15–32)	62.0 ± 11.4 (40–83)	<0.001
Onset-to-admission time (days)	30.0 (14.0, 60.0)	105.0 (30.0, 365.0)	<0.001
N_2_O exposure time(days)	183.0 (30.0, 547.5)	-	-
**Symptoms**			
Paresthesia	76.0%	79.2%	0.707
Weakness	52.0%	79.2%	0.005
Unsteady gait	34.0%	75.0%	<0.001
Urination and defecation disorders	16.0%	43.8%	0.003
Memory impairment	10.0%	6.3%	0.715
Involuntary movement	8.0%	4.2%	0.678
Anorexia	8.0%	29.2%	0.007
Hyperpigmentation	4.0%	4.2%	1.000
**Neurological exams**			
Loss of deep sense	52.0%	77.1%	0.010
Decreased muscle power	48.0%	68.8%	0.149
Decreased/Absence deep tendon reflex	44.0%	31.3%	0.193
Increased deep tendon reflex	8.0%	14.6%	0.302
Romberg sign(+)	14.0%	39.6%	0.004
Babinski sign(+)	10.0%	37.5%	0.001
FDRS-scores	3.0 (2.0, 5.0)	5.0 (4.0, 7.0)	<0.001
**Investigations**			
VB12 (180–914 pg/ml)	218.0 (121.0, 350.0)	96.0 (50.0, 170.0)	<0.001
VB12 deficiency	41.4%	80.5%	0.001
HCY (0–15 umol/L)	36.9 (16.5, 71.2)	40.1 (16.1, 114.0)	0.414
Hyper homocysteine	81.0%	80.0%	1.000
Megaloblastic anemia	31.6%	56.3%	0.022
**Electromyography**			
Positive rate	97.0%	84.6%	0.159
Aonal + demyelinating	66.7%	50.0%	0.286
Demyelinating	24.2%	30.8%	0.769
Aonal	6.1%	3.9%	1.000
**Spine MRI changes**			
Positive rate	80.0%	64.2%	0.034
Inverse V sign	72.0%	31.3%	<0.001
Involvement of thoracic cord	2.0%	16.7%	0.012
Number of spinal segments (segment)	5.0 (4.0, 6.0)	6.0 (5.0, 7.5)	<0.001
Anterior-posterior diameter (mm)	5.3 ± 0.8	4.2 ± 1.0	<0.001

*VB12, Vitamin B12; HCY, homocysteine; MRI, magnetic resonance imaging; +, positive*.

### Laboratory Tests

Approximately 80% of the patients in each group had an elevated homocysteine level, which was the most common laboratory abnormality. The patients with N_2_O-related SCD had a higher VB12 level than the patients with N_2_O-unrelated SCD (median: 218.0, IQR: 121.0–350.0 pg/mL vs. median: 96.0, IQR: 50.0–170.0 pg/mL, respectively; *P* < 0.01). A lower proportion of patients with N_2_O-related SCD than patients with N_2_O-unrelated SCD had VB12 deficiency (41.4 vs. 80.5%, respectively; *P* < 0.05) and megaloblastic anemia (31.6 vs. 56.3%, respectively; *P* < 0.05).

### MRI and EMG Findings

**Figure 2:**
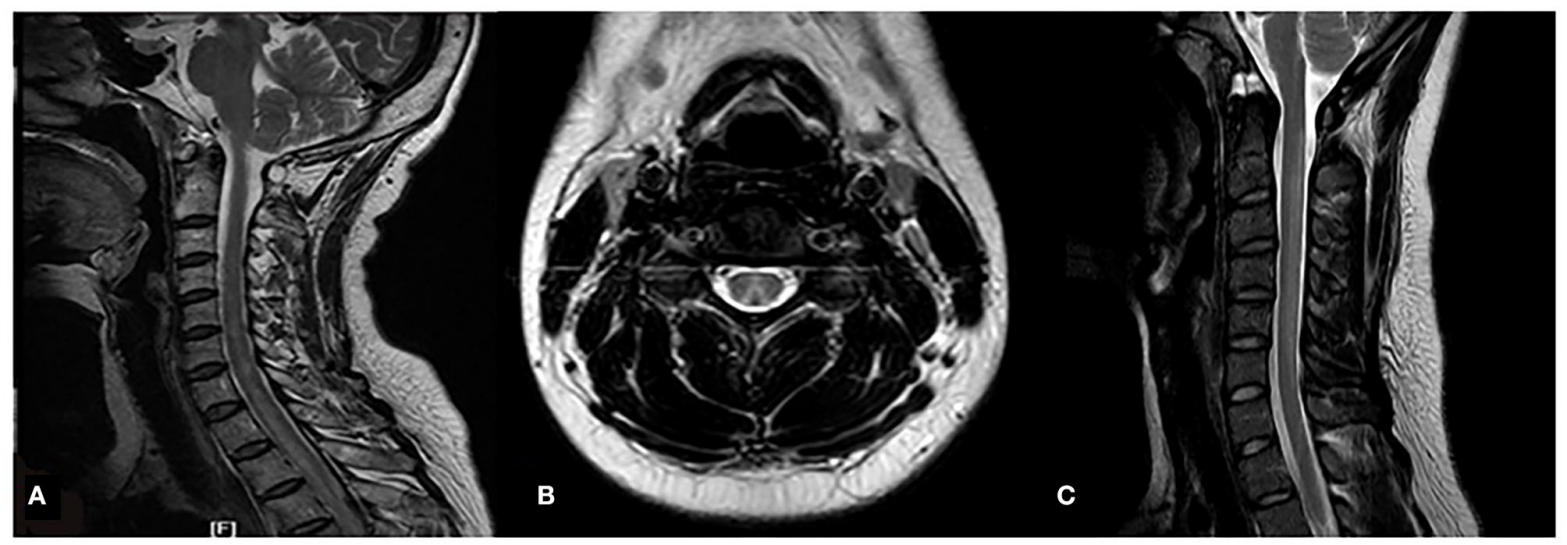
MRI images of the spinal lesions in the 2 patient groups. N_2_O-unrelated SCD patient; 60-year-old woman, T2WI sagittal high-signal intensity lesion extends from C2 to T3 (white arrow) **(A)**; N_2_O-related SCD patients, 23-year-old man, T2WI axial images of the lesions show typical inverted V sign **(B)**, T2WI sagittal high-signal intensity extends from C2-C6 (white arrow) **(C)**.

N_2_O-related SCD patients compared with the N_2_O-unrelated SCD patients had a higher positivity rate on spinal magnetic resonance imaging (MRI) (80.0 vs. 64.2%), wider spinal lesions on sagittal MRI (5.3 ± 0.8 mm vs. 4.2 ± 1.0 mm), fewer spinal segments with lesions (median: 5, IQR: 4–6 segments vs. median: 6, IQR: 5–7.5 segments), and higher rate of the inverted V sign on axial MRI (72.0 vs. 31.2%) (all *P* < 0.05) (Table 1 and Figure 2). The rates of abnormal electromyographs were elevated both groups, with no difference between the rates (N_2_O-related vs N_2_O-unrelated SCD, 97 vs. 85%, respectively; *P* > 0.05). The most common type of peripheral neuropathy was mixed axonal and demyelinating neuropathy.

## Discussion

SCD is a rare neurological disease caused by VB12 deficiency, which affects the posterior and lateral funiculus of the spinal cord. Its characteristic pathological manifestations are swelling of the myelin sheath and patchy spongy vacuolization (8, 9). Because the human body usually contains a large reserve of VB12 related to the enterohepatic circulation and renal reabsorption, the clinical signs/symptoms of SCD appear only after years of insufficient VB12 intake (10). Therefore, classical SCD is often secondary to gastrointestinal surgeries, a long-term vegetarian diet, or a chronic digestive disease such as atrophic gastritis (11, 12). Our current study found that the number of patients with SCD related to N_2_O use who were admitted to our hospital over the last 5 years was comparable to the number of patients with classical SCD, which indicates that recreational N_2_O use has become one of the main causes of SCD in China, especially in young patients. Although the definitive pathophysiological mechanisms involved in the development of SCD induced by N_2_O use are not entirely clear, the main mechanism is believed to be the irreversible oxidation of the cobalt atom of VB12 by N_2_O, which interferes with the methylation of the myelin protein, finally leading to myelinoclasis (13).

Classical SCD usually has a subacute or chronic onset, with the common clinical manifestations of symmetrical disturbance of deep sensation and spastic paralysis of the lower limbs. Some patients with classical SCD may also present with autonomic dysfunction and cognitive impairment (14, 15). Our study showed that the impairments of the spinal cords of the N_2_O-unrelated SCD patients were more serious than the impairments seen in the N_2_O-related SCD patients. We believe that, compared with the N_2_O-unrelated SCD patients, the younger age of the N_2_O-related SCD patients along with a shorter interval between the onset of disease and their admission to the hospital may contribute greatly to their less severe clinical manifestations.

Many studies have shown that patients with SCD related to N_2_O use have decreased serum VB12 levels and hyperhomocysteinemia (4, 16). Hyperhomocysteinemia is caused by VB12 deficiency since VB12 is an indispensable enzyme for homocysteine metabolism. However, recent studies have shown that the VB12 deficiency caused by N_2_O use is a functional deficiency instead of an absolute deficiency as suggested by the finding that blood tests for VB12 detect oxidized and inactivated VB12 (6). This finding is consistent with our study findings, where half of the N_2_O-related SCD patients had VB12 levels within the normal range; whereas most (81%) of the patients in this group had hyperhomocysteinemia. Therefore, the serum VB12 level is of limited value for the diagnosis of SCD induced by N_2_O inhalation, while an increase in homocysteine due to inactivated VB12 may be a more effective index. The percentage of N_2_O-unrelated SCD patients with megaloblastic anemia was significantly higher than the percentage of N_2_O-related SCD patients. This finding might be attributed to the longer duration of VB12 deficiency in the N_2_O-unrelated SCD patients, which exerted more severe effects on the hematological systems of these patients.

Classical SCD lesions show high-intensity signals on T2WI in the posterior and lateral funiculi of the spinal cord, which can present as an inverted V shape on axial MRI. The spinal lesions often involve the cervical spinal cord and the upper segments of the thoracic spinal cord (17, 18). Our study showed that unlike the lesions of N_2_O-unrelated SCD patients, the spinal lesions of N_2_O-related SCD patients shared the following characteristics: (1) lesions were usually localized to the cervical spinal cord and rarely extended to the thoracic spinal cord; (2) lesions were wider, which is a manifestation of swelling; and (3) the inverted V sign was more common. These findings might be related to the relatively acute process of spinal cord demyelination, which was due to the inhalation of a large amount of N_2_O over a short time and which was accompanied by an inflammation stage manifested by swelling of the spinal cord. Spinal demyelination induced by other factors such as a vegetarian diet is a chronic process with limited inflammatory changes. Thus, spinal MRI positivity rate of N_2_O-related SCD patients was significantly higher than the rate of N_2_O-unrelated SCD patients. Additionally, the age difference between the 2 patient groups also contributed to the difference between the MRI positivity rate. A study by Lan et al. showed that while only half of adult N_2_O-related SCD patients had abnormal MRI findings, almost all the adolescent N_2_O-related SCD patients had abnormal MRI changes (19). This may be attributed to the immature myelin sheaths in young people, which are vulnerable to pathological impairments.

Recent studies have revealed that nervous system impairments related to N_2_O use might not be completely due to compromised VB12 metabolism. For example, studies have reported finding patients with N_2_O-induced motor neuropathy and without VB12 deficiency (20). Currently, N_2_O alone is believed to cause neurotoxicity by overstimulation of the adrenergic and dopaminergic neurons, to inhibit the N-methyl-D-aspartate receptor, and to induce the oxidative stress response and ischemic injury (21, 22). These mechanisms involved in the development of neurotoxicity need further e exploration in order to develop new clinical therapies.

This study has limitations: First, this was a retrospective study with unavoidable selection bias. Second, there were various types of containers for recreational N_2_O, and therefore determining the dose of N_2_O inhaled by patients and the dose-effect relationship between N_2_O use and SCD could not be investigated. Third, since recreational N_2_O is an illegally manufactured product, with industrial N_2_O being frequently used for the recreational N_2_O products, we cannot rule out the possibility that the damage to the nervous systems of the study patients could have been related to other types of gases also present in the N_2_O products.

## Conclusion

In conclusion, the recreational use of N_2_O has become the major cause of SCD in young patients in China. Compared with classical SCD patients, the patients with SCD related to N_2_O use have less severe clinical presentations, a less obvious decrease in the serum VB12 levels, and an increased incidence of abnormal spinal MRI findings. Therefore, young patients with similar clinical manifestations and spinal MRI characteristics should be asked about their detailed exposure history to N_2_O.

## Data Availability Statement

The original contributions generated in the study are included in the article/supplementary material, further inquiries can be directed to the corresponding author.

## Ethics Statement

This study was approved by the Ethics Committee of Affiliated Shengjing Hospital of China Medical University. All patients or the patient's next-of-kin if a patient could not sign because of disability or underage provided written informed consent to participate. Written informed consent was obtained from the patients or from the patient's next-of-kin for publication of this research and any accompanying images.

## Author Contributions

HG, WL, and DZ: conceptualization. JR, YM, and XD: data collection, analysis, and investigation. HG and WL: writing (original draft). DZ: writing (review and editing). All authors approved the final version of the manuscript and agreed to be accountable for all aspects of the work.

## Conflict of Interest

The authors declare that the research was conducted in the absence of any commercial or financial relationships that could be construed as a potential conflict of interest.
